# Boosting tendon repair: interplay of cells, growth factors and scaffold-free and gel-based carriers

**DOI:** 10.1186/s40634-017-0117-1

**Published:** 2018-01-05

**Authors:** Zexing Yan, Heyong Yin, Michael Nerlich, Christian G. Pfeifer, Denitsa Docheva

**Affiliations:** 1 Laboratory of Experimental Trauma Surgery, Department of Trauma Surgery, University Regensburg Medical Centre, Franz-Josef-Strauss-Allee 11, 93053 Regensburg, Germany; 2 Director of Experimental Trauma Surgery, Department of Trauma Surgery, University Regensburg Medical Centre, Franz-Josef-Strauss-Allee 11, 93053 Regensburg, Germany

**Keywords:** Tendons, Tendon repair, Tendon tissue engineering, Adipose-derived mesenchymal stem cells, Bone marrow-derived mesenchymal stem cells, Tendon stem progenitor cells, Scaffolds, Hydrogels, Scaffold-free carriers, Cell sheets

## Abstract

**Background:**

Tendons are dense connective tissues and critical components for the integrity and function of the musculoskeletal system. Tendons connect bone to muscle and transmit forces on which locomotion entirely depends. Due to trauma, overuse and age-related degeneration, many people suffer from acute or chronic tendon injuries. Owing to their hypovascularity and hypocellularity, tendinopathies remain a substantial challenge for both clinicians and researchers. Surgical treatment includes suture or transplantation of autograft, allograft or xenograft, and these serve as the most common technique for rescuing tendon injuries. However, the therapeutic efficacies are limited by drawbacks including inevitable donor site morbidity, poor graft integration, adhesion formations and high rates of recurrent tearing. This review summarizes the literature of the past 10 y concerning scaffold-free and gel-based approaches for treating tendon injuries, with emphasis on specific advantages of such modes of application, as well as the obtained results regarding in vitro and in vivo tenogenesis.

**Results:**

The search was focused on publications released after 2006 and 83 articles have been analysed. The main results are summarizing and discussing the clear advantages of scaffold-free and hydrogels carriers that can be functionalized with cells alone or in combination with growth factors.

**Conclusion:**

The improved understanding of tissue resident adult stem cells has made a significant progress in recent years as well as strategies to steer their fate toward tendon lineage, with the help of growth factors, have been identified. The field of tendon tissue engineering is exploring diverse models spanning from hard scaffolds to gel-based and scaffold-free approaches seeking easier cell delivery and integration in the site of injury. Still, the field needs to consider a multifactorial approach that is based on the combination and fine-tuning of chemical and biomechanical stimuli. Taken together, tendon tissue engineering has now excellent foundations and enters the period of precision and translation to models with clinical relevance on which better treatment options of tendon injuries can be shaped up.

## Introduction

Tendons, which connect bone to muscle, are a crucial part of the locomotion system (Aslan et al. 2008). Tendons transmit forces from muscle to bone (Nourissat et al. 2015) and are able to withstand high tension and store elastic energy which in turn makes movements more ergonomic (Docheva et al. 2015). Tendons are very hierarchically organized tissues containing tendon-specific fibroblastic cells named according to their maturation state as tendon stem/progenitor cells (TSPCs), tenoblasts or tenocytes, the latter being the most terminally differentiated cells (Docheva et al. 2015). The cells are embedded in a three-dimensional network of extracellular matrix (ECM) consisting predominantly of type I collagen, other collagens (such type III and V), proteoglycans, elastin and fibronectin (Andres and Murrell 2008; Sayegh et al. 2015). The whole tendon unit and the tendon sub-units are wrapped with epitenon and endotenon loose connective sheets, respectively. It has been suggested that these sheets contain stem/progenitor-like cells (Docheva et al. 2015; Wu et al. 2017).

Tendon injuries are very common in trauma and orthopaedic surgery. Tendon injuries affect great range of patients from young to elderly patients, from workers to professional athletes (Docheva et al. 2015). Tendon healing follows a typical wound-healing course: inflammatory phase, proliferative phase, and then a remodelling phase (Voleti et al. 2012). The first short phase is characterized by the infiltration of inflammatory cells like platelets, monocytes, macrophages and neutrophils which release chemotactic agents activating and attracting tendon cells from the injured ends and tendon sheets. During the proliferative phase, the tendon fibroblasts start to proliferate and create abundant ECM. In the remodelling phase, the collagen fibres become parallel to the muscle force direction which is critical for the gain in tendon biomechanical strength (Evans 2012). In general, tendons have limited ability to repair as the initially formed scar tissue has inferior biomechanical properties compared to the original tendon tissue and if improperly replaced or remodelled it can be the foundation of increased rates of re-occurring ruptures.

**Fig. 1:**
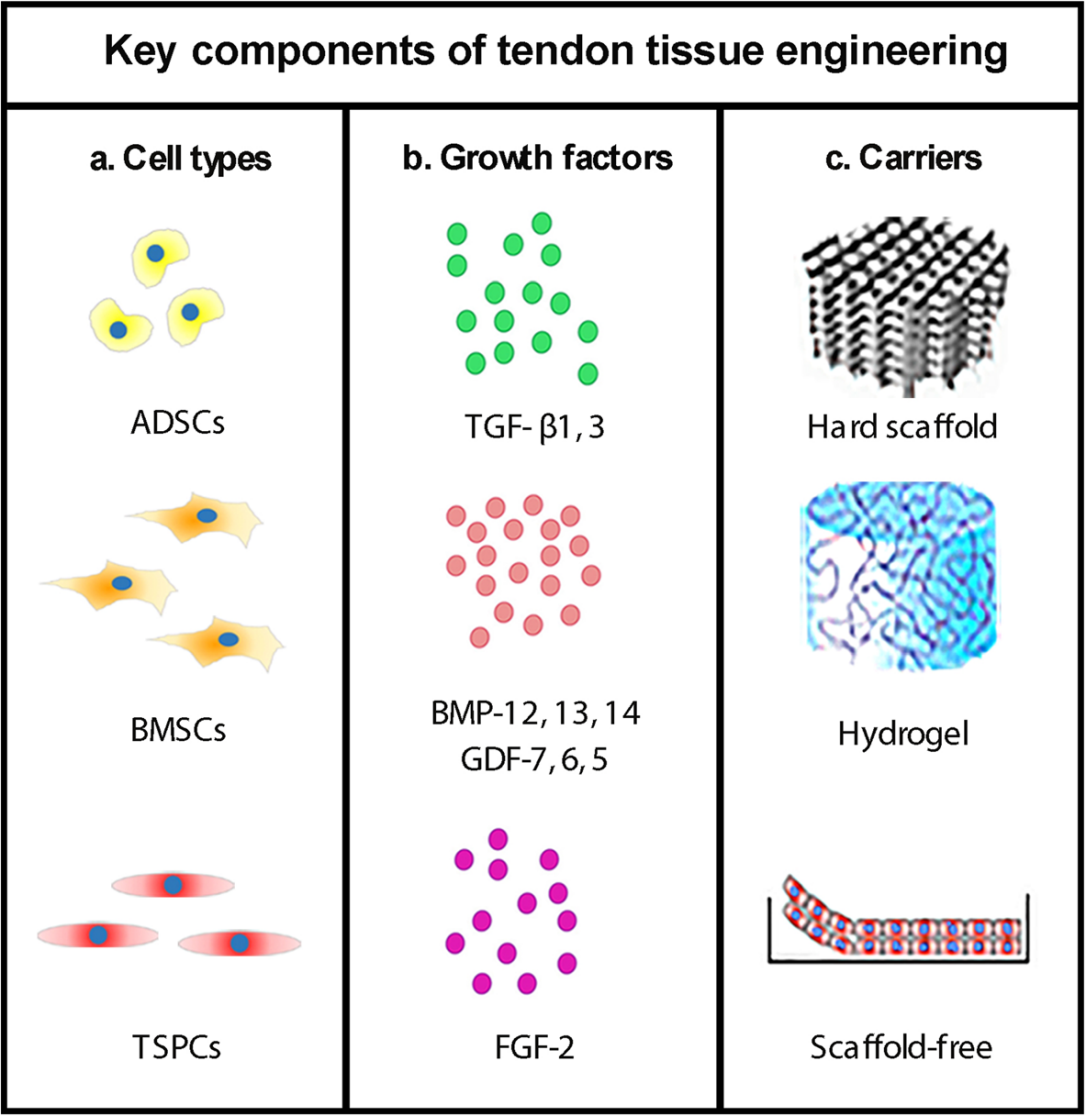
Summary of the key components of tendon tissue engineering. **a** Favourable cell types; **b** Pro-tenogenic growth factors; **c** Possible scaffolds and self-assembled materials. Various combinations between the components are possible

Due to the highlighted above functional limitations and high rates of re-injury of once rupture tendon, various surgical techniques including surgical suture, tendon autograft and allograft transfer for repairing tendon injuries have been already been vastly described (Oryan et al. 2014). However, the long-term clinical outcomes of surgical treatments are still not satisfactory. Thus, new and novel techniques need to be developed. The growing interest in non-operative and conservative treatment options, even for total tendon ruptures calls for new ways to initiate endogenous tissue repair by regenerative mechanisms. Tissue engineering is a promising alternative treatment for achieving complete recovery of ruptured tendons and in general, is based on the combination of reparative cells, growth factors and carriers (Fig. 1).

The aim of this review is to revise the recent literature about tendon tissue engineering with particular focus on two types of carriers, hydrogels and scaffold-free approaches. Since most reviews have focused on two of the three components of the tissue engineering approach, namely cell types and growth factors, the carriers (third component) have not often been the focus. Actually, the two types of carriers selected for evaluation in this review are particularly innovative and constitute a research field of increasing importance which can contribute to enhanced tendon tissue engineering.

The review constitutes of the following parts: (i) clinical relevance of tendon injury is provided; (ii) an overview of the areas involved in tendon tissue engineering (cells, growth factors, carriers) is given; (iii) the current status on cell types and growth factors is briefly summarized; and (iv) a detailed information on carriers with particular focus on hydrogels and scaffold-free options is delivered and discussed.

**Fig. 2:**
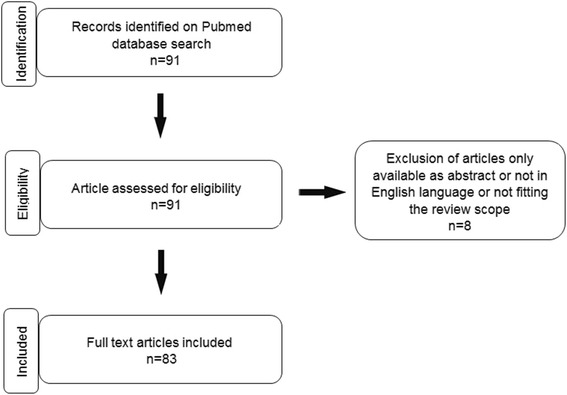
Flow chart of the search strategy and article selection in this review

A computerized search of potentially eligible studies was performed in PubMed and the date of the last search was October 30, 2017. Database search and key inclusion criteria followed the terms “tendon” AND “tendon injury” AND “tendon repair” AND “tendon tissue engineering” AND “mesenchymal stem cells” AND “growth factors” AND “scaffold-free” OR “gel-based” OR “cell sheet”. The search was focused to publications (both forms none- and open-access) released after 2006. Articles available only as abstract or not in English language or not fitting the review scope were excluded. A summary of the search strategy and article selection in this review is shown in Fig. 2.

## Review

### Tendon clinical relevance

Tendon injuries are usually induced by intrinsic (age, gender, weight, metabolic diseases) and extrinsic (sport injury, overload and occupation) factors. To date, treating tendon injuries costs healthcare providers in USA 30 billion dollars, and in Europe over 115 billion euros per year (Abbah et al. 2014). It is reported that 30–50% of sport injuries include tendon component (September et al. 2007). Epidemiologic reports indicate that annually over 4.4 million patient clinical visits in USA are due to shoulder maladies associated with tendon disorders (Nixon et al. 2012). Chronic and acute injuries can occur in any tendon, although, the most frequently affected tendons are Achilles and rotator cuff tendons. Raikin et al. 2013 reviewed that regarding Achilles tendon injuries 76% of the ruptures are acute with average patient age of 46.4 years, whereas 24% are chronic. Rotator cuff tears affect up to 50% of patients older than 50 years and are a common cause of function-limiting pain and weakness of the shoulder (Isaac et al. 2012). Tendon repair after injury is extremely poor and inefficient because of the low cellularity, vascularity and metabolic activity of the tendon tissue (Liu et al. 2011; Thangarajah et al. 2015). Moreover, in most patients, especially in aged individuals, the healed tendon usually does not regain the mechanical properties of the uninjured tissue. As a consequence, the tendon thickens and stiffens to overcome the lower unit mechanical strength and, hence, the tendon quality and its functional activity are inferior to that of healthy tendon (Docheva et al. 2015). For the above reasons identifying and designing strategies to augment tendon healing are of very high relevance. Over the years, the tendon scientific community has particularly focused on establishing and improving various tendon tissue engineering models which become very promising for achieving a breakthrough in tendon injury management.

As previously mentioned, the most common strategy for restoring ruptured tendons is surgical repair which at times does not result in complete and satisfactory tendon healing. Especially in elderly individuals, surgical repair shows poor long-term outcomes due to re-rupture, restrictive adhesions and suboptimal strength and functionality linked to decreased biomechanical properties (Myer and Fowler 2016). Considerable side effects and complications are seen with operative treatment. In Achilles tendon repair, the majority of patients occur in men aged 30–49 and are reported due to wound and tendon healing problems, like re-rupture, tissue damage and necrosis and subsequently wound infection (Raikin et al. 2013; Thomopoulos et al. 2015). It has been known that several factors, including advanced patient age, large tear size, severe muscle atrophy and fatty infiltration, systemic metabolic diseases and smoking are associated with failed or poor tendon healing (Montgomery et al. 2012). Since traditional surgical options present limitations and complications, non-operative treatment has gained interest in recent years. The challenge of non-operative treatment is the diastasis of the ruptured tendon ends. Any gap and tendon defect may delay the healing process and may result in an insufficient tendinous tissue. Therefore, innovative treatment options such as tissue engineering of ruptured tendons has drawn great interest and driven multi-centre experimental and pre-clinical research to solve this issue.

### Tendon tissue engineering

Tissue engineering aims to induce and support tissue self-repair or to produce a functional tissue replacement in vitro that is subsequently implanted in vivo at the site of injury (Youngstrom and Barrett 2016). Hence, tissue engineering may play a major role in improving the management of tendon injuries through grafting engineered tendon segment at the site of rupture (Hsieh et al. 2016b; Yin et al. 2016). We have previously reviewed in great detail (Docheva et al. 2015; Wu et al. 2017) on the two main components of tendon tissue engineering namely cell types and growth factors as well as given summary on the recent advancment of natural hard scaffolds. The current review focuses on the “third component”, the carrier and in particular on hydrogels and scaffold-free approaches. A hydrogel is a network of natural or synthetic polymer chains with high content of liquid. Due to their significant water content, hydrogels are softer and easier to mould and therefore suitable as filling materials. On the other hand, scaffold-free approaches allow the cells to form natural cell to cell and cell to matrix connections without artificial interference and with firing of specific molecular signalling events that can trigger lineage mantainance or even facilitate further maturation.

### Cell types

In tendon tissue engineering, mainly stem or progenitor cells of mesenchymal origin are being implemented to create tendon grafts and support graft incorporation (Andres and Murrell 2008). Several cell types have been more favourable and these belong to the mesenchymal stem cells family (MSCs) found in different tissue sources. MSCs extracted from adipose tissues or the bone marrow or tendon-derived cells, including local MSC-related but distinct TSPCs, have been suggested as the most suitable cell types (Yin et al. 2016). These cell types have clear advantages like differentiation potential as well as paracrine effects, which have been reported to play a crucial role in their beneficial properties, by promoting angiogenesis, stimulating local progenitor and mature cells, or regulating inflammation and immune cell functions (da Silva et al. 2009).

MSCs derived from adipose tissue (ADSCs) are an attractive candidate cell type due to their easy isolation, multi-potentiality and high responsiveness to distinct environment cues (Zarychta-Wisniewska et al. 2017). Adipose tissue is abundant in human and animals and subcutaneous adipose aspirate can be easily harvested by a minimally invasive procedure (Deng et al. 2014). However, the main disadvantage of ADSCs is their preference towards adipogenesis in vivo (Neo et al. 2016).

Bone marrow mesenchymal stem cells (BMSCs) due to being best characterized are the most widely used stem cell type. One recent study showed that BMSCs are more responsive to bone morphogenetic protein-12 (BMP-12) stimulation and hence exhibited superior tenogenic differentiation capacity when compared to ADSCs (Dai et al. 2015). However, BMSCs also have some limitations, such as painful harvesting procedure with frequently low cell yield, reduced MSC quality with advanced donor age (Zhao et al. 2009), ectopic ossification and higher risk of adhesion formation when transplanted in vivo (Hsieh et al. 2016a).

TSPCs are a cell type that moved in the research spotlight due to their inherent pro-tenogenic abilities. They were first reported and described in 2007(Bi et al. 2007) and subsequently identified in different tendons, isolated from different species and characterized to some extent (Kohler et al. 2013). One clear advantage of TSPCs is their greater potential for tenogenesis. TSPCs express higher mRNA levels of tendon-related gene markers including the transcription factor Scleraxis (Scx) and the late differentiation factor Tenomodulin (Tnmd) than BMSCs (Ni et al. 2013). Furthermore, we have recently shown in two consecutive studies that native or genetically induced TSPCs transplanted in clinically relevant Achilles tendon defect model in *Rattus norvegicus* are superior to BMSCs as TSPCs grafting resulted in advanced, significantly less ossified and more mature ECM of the tendon at the remodelling phase of the healing process (Yin et al. 2013). However, TSPCs hold one main disadvantage namely their isolation that is associated with many limitations and co-morbidity. One strategy to overcome this difficulty is to use ADSCs or BMSCs that have been pre-differentiated towards the tendon lineage with the help of growth factors, a topic we will discuss in the next chapter and in Table 1.

**Table 1:** Pro-tenogenic growth factors

Growth factor	Cell source	Cell proliferation and differentiation	Gene expression	ECM production	Study type and animal model	Reference
TGF-β1 (5 ng/ml) & TNF-α (0.0025 ng/ml)	Rat TSPCs	TGF- β1 or TNF-α alone did not enhance the proliferation and differentiation of TSPCs, but in combination or upon sequential application of these two signalling molecules facilitated their proliferation and differentiation. Furthermore the combined application of TGF-β1 in addition to TNF-α could resque the growth inhibition induced by TNF-a.	TGF-β signalling pathway significantly activated the expression levels of certain members of Smad family. In addition, the expression of tenogenic/osteogenic markers was also significantly increased under the combined treatment of TGF-β1 and TNF-α	Not studied	In vitro	Han et al. 2017
TGF-β3 (20 ng/ml)	Equine embryo-derived SCs (ESCs)	TGF- β3 can promote tenocyte differentiation of ESCs in 2D monolayer cultures. The ESCs did not develop areas of cartilage or bone tissue, and it was concluded that the differentiation response is specific to tenogenic lineage.	Express tendon-associated genes were detected. The presence of TGF-β3 induced the expression of late-onset tenogenic markers, namely Tnmd and thrombospondin 4, which were not detected in untreated cultures over the early time course.	ESCs treated with TGF- β3 organized a tendon-like matrix without evidence of bone or cartilage formation.	In vitro	Barsby et al. 2014
GDF-5 (BMP-14) (100 ng/ml)	Rat ADSCs	GDF-5 led to increased ADSCs proliferation in a dose- and time-dependent manner. In the time kinetic studies, the proliferation rate of ADSCs treated with 100 ng/ml of GDF-5 increased significantly at all time points.	ADSCs demonstrated enhanced ECM production and tenogenic marker gene expression that was increased with longer exposure. GDF-5 also altered the expression of ECM remodelling genes, with no specific dose and time trends observed. The two key tenogenic markers Scx and Tnmd showed clear upregulation with 100 ng/ml GDF-5.	Col I expression increased in cells treated with 100 ng/ml of GDF-5 compared to control. No significant difference was found for Col III.	In vitro	Park et al. 2010
GDF-5 (BMP-14) (0,5,25,50,100 ng/ml)	Human BMSCs	GDF-5 did not alter the proliferation rate significantly. The use of GDF-5 induced tenogenic differentiation of this cell type without effect on cell doubling. It appears that GDF-5 at a concentration of 100 ng/ml provides the most optimal cell phenotypic response.	The tenogenic marker genes Scx and TnC were upregulated at day 4 after GDF-5 treatment. However, at day 7, only Scx was persistently upregulated, the expression of Runx2 and Sox9 genes were significantly downregulated. In conclusion this growth factor augmented the levels tenogenic marker genes and downregulated non-tenogenic marker gene expression.	There were no significant differences in total collagen deposition between GDF-5 treated groups with different concentration levels. However to non-treated controls it augmented the total collagen amount.	In vitro	Tan et al. 2012
GDF-6 (BMP-13) (20 ng/ml)	Rabbit BMSCs	Cell proliferation was not studied. BMSCs differentiation into tenocytes was studied via gene expression.	Expression of Scx and Tnmd was significantly higher under GDF-6 stimulation. Expression levels of TnC and Col I were higher in the control group but not significant.	Histological evaluation of patellar tendon injury repair model suggested that transplantation of GDF-6-treated BMSCs improved tendon healing due to increase Col deposition and presence of more organized Col fibers.	In vitro and in vivo rat model	Jiang et al. 2016
GDF-7 (BMP-12) (50 ng/ml)	Equine BMSCs; in vitro	Cell proliferation was not studied Equine BMSCs defined by their expression of markers such as Oct4, Sox-2 and Nanog, have the capability to differentiate in tenocytes based on gene expression.	Following exposure to BMP-12 the BMSCs upregulated the expression of two tendon-related markers, Tnmd and decorin.	Not studied.	In vitro	Violini et al. 2009
BMP-12 (GDF-7) (50 or 100 ng/)	Human ADSCs; in vitro	There was no significant difference in proliferation rates of ADSCs after treatment with BMP-12, regardless of the applied doses. BMP-12 activated tenogenesis of ADSCs based on gene expression analyses.	ADSCs treated with BMP-12 for 7 days resulted in up-regulation of tenogeinic genes, such as Scx and Mohawk but also Runx2, an osteogenic maker gene was elevated.	BMP-12 treatment increased expression of Col I in ADSCs.	In vitro	Zarychta-Wisniewska et al. 2017
FGF-2 (5 μg/ml)	Rat TSPCs	In vivo evaluation at 2 and 4 weeks post-operation showed that the FGF-2-treated group has greater numbers of cells in the granulation tissue than the control group. At 6 weeks there was no significant difference in cell number between the FGF-2-treated group and the control group.	The expression level of Scx increased in the FGF-2-treated group from 4 to 8 weeks, and Tnmd levels increased significantly from 4 to 12 weeks postoperatively. Sox9 expression was significantly up-regulated at 4 weeks in the FGF-2-treated group.	Not studied.	In vivo; rat rotator cuff healing model	Tokunaga et al. 2015

### Growth factors

Growth factors play an important role in tendon tissue engineering. They are peptide signalling molecules with a dominant biological role in regulating cell proliferation and differentiation (Branford et al. 2014). Growth factors relevant to the tendon healing process and MSC tenogenesis include families such as bone morphogenetic protein (BMP) family, fibroblast growth factor (FGF), transforming growth factor beta (TGF-β), insulin-like growth factor (IGF), vascular endothelial growth factor (VEGF), connective tissue growth factor (CTGF) and platelet-derived growth factor (PDGF). Many studies have shown that MSCs are very sensitive to the above factors and they can influence their stemness and steer the rate of proliferation and extent of terminal differentiation (Barsby et al. 2014; Halper 2014; Han et al. 2017; Jiang et al. 2016; Lui et al. 2016; Park et al. 2010; Tan et al. 2012; Tokunaga et al. 2015; Violini et al. 2009; Zarychta-Wisniewska et al. 2017). However, only some of the growth factors have promising effects on MSC tenogenesis and example studies and their outcomes are described in Table 1.

Growth factor stimulation protocols of MSCs to achieve tenogenesis in vitro are advancing, However, in order to become very efficient and reproducible, further research understanding the exact molecular signalling events orchestrating and controlling the step-wise commitment process is required. In this respect, great knowledge can be obtained from tendon developmental studies based on gene knockout and reporter mouse strains (Dex et al. 2016). Another research area that can lead to accelerated tenogenesis is applying mechanical loading in combination with growth factors (James et al. 2008).

### Carriers

#### Hard scaffolds

Scaffolds are used to deliver cells and drugs into the body (Garg et al. 2012; Turner and Badylak 2013). Classically, scaffolds are made of hard materials displaying distinct architecture, porosity, interconnectivity, large surface and biocompatibility. The current materials used in tendon tissue engineering have provided significant advances in structural integrity and biological compatibility and in many cases the results are superior to those observed in natural healing (Butler et al. 2008; Liu et al. 2008; Sahoo et al. 2010b). Several kinds of scaffolds have been widely used in tendon tissue engineering such as biologically-based or synthetic scaffolds (Youngstrom and Barrett 2016). Biological scaffolds which includes dermis, pericardium, small intestine submucosa, and tendon, are composed of natural collagen fibres that are bioactive and beneficial for cell attachment, viability and proliferation (Woon et al. 2011). The structure of biological scaffolds may better resemble the original tissue and be more feasible for incorporating and supporting cells but they could be more difficult to obtain and might require additional and more complicated surgery in order to be delivered into the site of injury. In addition, to avoid in vivo immunoreaction, biological scaffolds need to be decellularized prior to implantation. Furthermore, another widely used scaffold type is silk, which has been shown to perform well both in vitro and in vivo tendon studies (Chen et al. 2010). Some other natural materials e.g. human umbilical veins and hyaluronic acid-based scaffolds have been also explored and given promising results in tendon tissue engineering (Fan et al. 2014; Hofmann et al. 2008).

Synthetic scaffolds made from polymers, such as polylactic acid (PLA); poly-L-lactid acid (PLLA); polyglycolic acid (PGA); poly-D,L-lactic-co-glycolic acid (PLGA); polyuria (PU) and poly-caprolactone (PCL), aim at mimicking the native tissue properties and are frequently produced by electrospinning technology (Sahoo et al. 2010b; Sahoo et al. 2010c). TSPCs cultivated on electrospun nanofibers showed augmented tenogenic gene expression (Yin et al. 2010) and increased ECM production (Xu et al. 2014). Some scaffolds are comprised of orientated nano/microfibers which results in similar structure to the native tendon tissue, hence enabling the typical spindle morphology of tendon cells and providing structural cues for tenogenesis (Sahoo et al. 2010). However, traditional hard scaffolds have limitations including poor cell seeding and distribution, low cell adhesion especially onto synthetic materials, unsatisfactory cell proliferation and differentiation and in some cases poor biocompatibility, low biodegradability, and non-matching to the tendon biomechanical properties (Liu et al. 2008; Lui et al. 2014; Ricchetti et al. 2012). The main difficulty arises from the structural and biomechanical properties of the materials and the creation of continuity when implanted in vivo. Research has suggested that materials resembling the hierarchical ECM organisation and dimensions, as well as the elastic properties of tendon tissues are preferable (Bagnaninchi et al. 2007). Forthcoming studies should focus on investigating their further optimization and long-term behaviour in clinically relevant models of tendon injury.

#### Hydrogels

As mentioned previously hydrogels are a network of natural or synthetic polymer chains with significant water content, possessing a higher degree of moulding. They are commonly used in tissue engineering because of their easier handling and good biocompatibility (Yamada et al. 2007). Hydrogels can fill up various defect shapes and can reach deeper into tissue injury site by percutaneous injection with minimal invasion and low side effects which normally occur after more complicated surgery techniques when hard scaffolds are used. Furthermore, hydrogels can incorporate various cells, drugs, and growth factors through simple mixing. Adherent cells can deposit in their vicinities, natural ECM and organise it appropriately and create a niche that responds to chemical and biomechanical stimuli. Hydrogels composed of natural biomaterials such as collagen, fibrin, hyaluronic acid, alginate, and other ECM proteins are most frequently used. In Table 2**,** we have summarized studies which focused on the application of hydrogels functionalized with cells for tendon tissue engineering. Most of the studies have investigated how such hydrogels affect the biological features of MSCs and tendon-derived cells in vitro (Annabi et al. 2014; Bian et al. 2013). Only few studies have tested the repair potential of hydrogels alone or in combination with cells in tendon injury models in vivo (Liu et al. 2008; Shah and Federoff 2011). Type I collagen and fibrin gels have been extensively studied to create tissue engineering constructs in vitro (Sander et al. 2011). Breidenbach et al. showed that fibrin gels loaded with TSPCs exhibit improved biological, structural, and mechanical characteristics compared with TSPCs-collagen gels in vitro (Breidenbach et al. 2015). Degen et al., Li et al., and Lopiz et al. reported that the application of hyaluronic acid or alginate hydrogels resulted in enhanced biomechanical and histological properties of the tendon repair tissue in vivo compared with control groups (Degen et al. 2016; Li et al. 2016; Lopiz et al. 2017). Farnebo et al., 2014 developed a novel injectable thermosensitive hydrogel derived from tendon ECM and seeded it with ADSCs (Farnebo et al. 2014). It formed a solid gel at body temperature and had good compatibility and support of cell adhesion and proliferation. Chiou et al., 2015 showed that the hydrogels combined with ADSCs augmented the tendon healing process in a rat injury model (Chiou et al. 2015). The above studies indicated that hydrogels hold a great potential for tissue engineering as they can provide a three-dimensional environment and can serve as an easier to handle cell delivery vehicle for surgical implantation. Despite several advantages and some promising experimental outcomes of the gel-based tissue engineering approach, one very critical limitation especially with regards to tendon repair is that the hydrogels cannot provide in a full tear scenario, the desired biomechanical strength and tissue continuity. Hence, their application can be specialized for cell or drug delivery in partial tendon lesions, contained tendon defects or underneath tendon sheets (Garg et al. 2012).

**Table 2:** Hydrogel-based studies on tendon tissue engineering

Hydrogel	Cell type	Cell proliferation and vitality	Gene expression and ECM	Biomechanical analyses	Study type, animal species and delivery method	Reference
Collagen/Fibrin	TSPCs	Not studied	TSPCs in the fibrin hydrogel exhibited significant upregulation of tenogenic markers (Scx, TnC, and F-mod) in comparison to Col gel. Tissue engineering constructs based on fibrin with TSPCs showed better collagen alignment compared to Col hydrogel.	Tissue engineered construct based on fibrin hydrogel showed higher linear stiffness than Col gel at day 10. However, no significant difference was detected at day 14.	In vitro	Breidenbach et al. 2015
Fibrin	BMSCs	Over 90% of labeled BMSCs remained viable after mixing in the fibrin hydrogel.	BMSCs continued to express the original phenotypic profile. Notably, all cells showed an absence of CD14, CD34, and CD45 expression. In addition, they maintained expression of CD105, CD73, and CD90.	At 2 weeks, there was a significant increase in stiffness of repaired tissue in the cell-treated group compared with the control group. However, at 4 weeks, this effect dissipated because both groups showed similar stiffness.	Athymic rat; Surgery	Degen et al. 2016
Fibrin	TSPCs	The cell proliferation rate in the TSPCs group treated with CTGF and ascorbic acid was lower compared with control group.	Not studied.	The transplantation of TSPC-fibrin constructs promoted tendon repair up to week 16, while TSPC that were pre-treated with CTGF showed better results already at 8. Both the ultimate stress and maximum Young’s modulus increased at a faster rate in the CTGF- treated TSPC group compared with the untreated group.	In vitro; Rat; Surgery	Lui et al. 2016
HA	Tendon fibroblasts	HA significantly decreased cell proliferation in a dose-dependent manner.	Immunofluorescence cytochemistry detected constitutive binding of HA and CD44 receptor on the tendon-derived cells. The expression levels of pro-collagen I α1 was not significantly decreased, but, the expression of procollagen III α1 was decreased significantly in a dose-dependent manner.	Not studied.	In vitro	Yamada et al. 2007
Tendon ECM	ADSCs	Spindle shaped cells were observed both on the gel surface as well as within the gels, with a homogenous distribution of cells throughout the gel.	Gene expression was not studied. This ECM gel solution can be delivered percutaneously into the zone of tendon injury in a rat model. After injection, the thermos-responsive behaviour of the ECM solution will allow it to gelate at body temperature. A supportive nanostructure of collagen fibres can be established to fit the three-dimensional space of the defect.	Not studied.	Rat; Injection	Farnebo et al. 2014
Tendon ECM	ADSCs	Proliferation rate of ADSC in tendon ECM-derived hydrogel treated with PRP was higher than untreated group.	Gene expression was not studied. Upon histological analysis, Hematoxylin and Eosin staining showed increased extracellular matrix formation in groups containing PRP and increased cellularity in groups containing ADSCs.	Mean ultimate failure load was increased in hydrogels augmented with PRP group at 2 weeks. At 4 weeks, hydrogel alone reached a similar mean ultimate failure load to hydrogels augmented with PRP and ADSCs. However, at 8 weeks, hydrogels with PRP and ADSCs demonstrated increased strength over other groups. In conclusion, groups containing both PRP and ASCs encouraged earlier mechanical strength and functional restoration.	Rat; Surgery	Chiou et al. 2015

#### Scaffold-free approaches

**Fig. 3:**
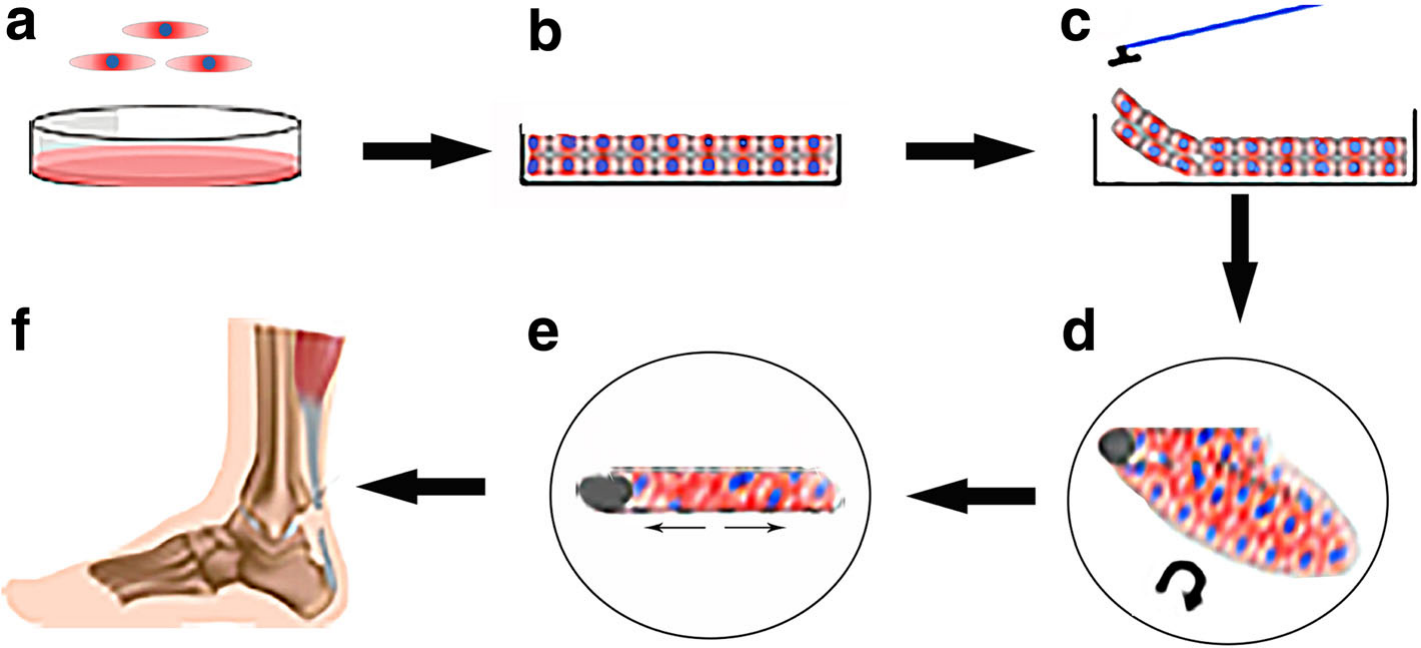
Cartoon of the procedure to form tendon-like cell sheet. **a** MSCs are plated in cell culture dish; **b** Cell monolayers are formed; **c** and **d** The monolayer is scraped out from dish surface and rolled up into a cell sheet; **e** and **f** The three-dimensional cell sheet is cultivated under static tension and let to mature prior transplantation in vivo

A scaffold-free approach means that cell form naturally the connections between each other and the matrix, thus tendon tissue engineered construct can be fabricated without the use of any carrier (Ni et al. 2013). With additional growth factors or mechanical stimuli, the cells can enhance the tenogenic differentiation process as well as the production of tendon-specific extracellular matrix (Kim et al. 2011). As human embryos can organize, proliferate and differentiate within itself without association to a scaffold so tendon tissue can be formed by a self-assembly process. Owaki et al. 2014 proved that TSPCs can produce scaffold-free tendon-like micro tissue (Owaki et al. 2014). This could be a powerful new approach for tendon tissue engineering. In chondogenesis, MSC pellet culture models are vastly used in vitro as well as implanted in vivo. MSCs are first condensed by centrifugation to form a pellet that in the presence of TGF-βand additional molecular factors undergo in the course of approximately 3 weeks into chondrogenesis, thus replicating key assets of embryonic cartilage formation (Mueller et al. 2013; Mueller et al. 2010). In tenogenesis, the so called cell sheet technology has been preferred (Markway et al. 2013). Cell sheets can eliminate the need for natural or synthetic carriers, thus avoiding the above listed disadvantages, as well as biocompatibility (Lui et al. 2014). A cartoon model of cell sheet formation is depicted in Fig. 3. Following cell seeding and upon reaching full confluency cells establish strong cell-to-cell contacts and produce large amounts of native ECM in their apical proximity. This allows an easy dissociation from the culture dish as a continuous integral cell layer. The layer can then be rolled up and subjected to static tension for a desired period of time. In Table 3**,** we have provided examples of studies dealing with cell sheets for tendon tissue engineering and their outcomes. The cell sheet technology can deliver cells to tendon and tendon-bone interface to accelerate tendon healing (Inagaki et al. 2013). One research group reported the use of anterior cruciate ligament (ACL)-derived CD34+ cell sheet that was wrapped around a tendon graft for ACL reconstruction in a rat model and concluded that the cell sheet augmented graft resulted in improved and developed a more mature bone-tendon healing (Mifune et al. 2013). One study transplanted TSPC sheets in a patellar tendon window injury model and reported that 2–6 weeks post-surgery the tendon healing was significantly promoted (Lui et al. 2014). Another study indicated that the use of a graft composed of multipotent stem cell sheets led to satisfactory reconstruction of complete musculotendinous junction rupture (Inagaki et al. 2013). Komatsu et al., 2016 proved that TSPC sheets significantly improved histological properties and collagen content at both 2 and 4 weeks after implantation into a rat Achilles tendon injury model, indicating that such an approach may effectively promote the early stages of tendon healing (Komatsu et al. 2016). One clear advantage of the cell sheet technology is the formation of native cell-to-cell and cell-to-matrix interactions which initiate the appropriate and inherent cell signalling cascades of the used cell types (Hashimoto et al. 2016; Mifune et al. 2013; Neo et al. 2016). Further application of growth factors, media supplements affecting the cell anabolism or mechanical stimuli can further boost the effectiveness of the cell sheet maturation towards enhanced in vitro tenogenesis (Tan et al. 2012; Violini et al. 2009). Follow up research is required to optimize the current protocols. For example as reported in Table 3, at present different protocols are used to form the cell sheets and there is not only one standard technique, thus further warranting investigations to optimize the cell sheet procedure. Another point for improvement is to find strategies to augment the mechanical properties of the cell sheet grafts prior to implantation in vitro which is mainly dependant on produced ECM amount and maturity level, as well as on the graft dimensions.

**Table 3:** Examples of tendon cell sheet models

Experimental cell sheet	Outcome	Preparation of cell sheet	Study type and animal models	Reference
Rabbit ADSCs sheet	Cell sheets were cultured over 3 weeks, and cell metabolic activity, cell sheet thickness, and early differentiation gene expression were analyzed. One week-old cell sheets displayed upregulation of early differentiation gene markers (Runx and Sox9). Cell sheet thickness and cell metabolic activity increased in the second and third week.	ADSCs were cultured in 6-well culture plates until 100% confluence. Confluent cells were then cultured in expansion medium supplemented with 50 mg/ml ascorbic acid for 3 weeks to facilitate cell sheet formation.	In vitro	Neo et al. 2016
Mouse MSC sheet	MSC sheet transplantation into musculotendinous junction at 4–8 weeks showed similar recovery of muscle mass and tension to the contralateral non-transplanted side. However, at 14–18 weeks, MSC sheet-treated group showed increased recovery of muscle mass and tension output. Engrafted MSCs primarily formed connective tissues and muscle fibres, and bridged the ruptured tendon-muscle fibre units.	The cells reached full confluence, detached from culture dishes with 2 mM EDTA, then collected and centrifuged into hybrid sheet/pellet like structure.	In vitro and in vivo; Mouse musculo-tendinous junction model	Hashimoto et al. 2016
Human ACL-derived CD34+ cell sheet	ACL-derived CD34+ cell sheet improved the ACL repair which was judged by histological assessment at week 2 and biomechanical evaluation at week 8 in a rat ACL injury model.	Cells were plated in temperature-responsive culture dishes at 37 °C for 17 h, and then incubated at 20 °C for 20 min, and afterwards the cell sheets detached spontaneously.	Rat ACL injury model	Mifune et al. 2013
Human rotator cuff-derived cell sheet	The cell sheets transplanted to the infraspinatus injury site induced angiogenesis and Col synthesis, and improved tendon-bone junction repair at 4 and 8 weeks postoperation.	Cells were cultured on 24-well temperature-responsive culture dishes at 37 °C for 17 h. Then, the plates were placed at room temperature for 20 min, and the cell sheets detached from the wells spontaneously.	Rat rotator cuff injury model	Harada et al. 2017
Rat TSPC GFP-labelled sheet	The TSPC sheet radiographically, histologically and biomechanically improved ACL healing in a rat model at week 2, 6 and 12 postoperatively. GFP-labelled TSPCs were detected at the graft-bone tunnel interface and in the intra-articular graft midsubstance in all samples at week 2.	Cells were plated in normal culture dishes in low-glucose medium. After 100% confluence, cell sheet was detached by rinsing with saline.	Rat ACL injury model	Lui et al. 2014
Rat TSPC sheet	TSPC sheet grafting into Achilles tendon defect significantly improved the histological features and Col content both at 2 and 4 weeks post-surgery, indicating that TSPC sheets can speed up tendon remodelling in the early stages of the healing process.	TSPC sheets were prepared by plating on temperature-responsive culture dishes. Cells were cultured for 3 days and then induced for cell sheet formation by treating with 25 mM ascorbic acid in complete culture medium at 37 °C. After 9 days, monolayer cell sheets were obtained by reducing the temperature from 37 °C to 20 °C for 20 min.	Rat Achilles tendon injury model	Komatsu, et al. 2016

## Conclusion

From hard scaffolds to gel-based and scaffold-free approaches, tendon tissue engineering has significantly progressed in recent years. The improved understanding of tissue resident adult stem cells, such as BMSCs, ADSCs and TSPCs, has been very helpful and a large number of studies have clarified the advantages and disadvantages of these cell types. Growth factors steering stem cell fate toward the tenogenic lineage have been identified and overall, the protocols for in vitro tenogenesis have been improved. Still, the field needs to consider a multifactorial approach that is based on the combination and fine-tuning of chemical and biomechanical stimuli in order to obtain optimal tenogenesis in vitro and in vivo. The field also has to move out of a ‘one size fits all’ strategy for treating tendon injuries and consider that different tendon defects can be treated by ‘custom design’ combination of cells and carriers and personalised physiotherapy. In particular, carrier-free and gel-based applications, in combination with autologous cells, can be very attractive option to enhance conservative treated tendon injuries as they can be delivered with minimal invasive operation procedure and may lead to quicker and better outcome. All in all, tendon tissue engineering has now excellent foundations and enters the period of precision and translation to models with clinical relevance and we think undoubtedly it will remain the most promising step forward for better treatment of tendon injuries.

## Abbreviations

ACL: Anterior cruciate ligament
ADSC: Adipose-derived stem cell
BMP: Bone morphogenetic protein
BMSC: Bone marrow stem cell
CTGF: Connective tissue growth factor
ECM: Extracellular matrix
ESCs: Equine embryo-derived stem cells
FGF: Fibroblast growth factor
F-mod: Fibromodulin
GDF: Growth differentiation factor
GFP: Green fluorescence protein
HA: Hyaluronic acid
IGF: Insulin growth like factor
PCL: Poly-caprolactone
PDGF: Platelet-derived growth factor
PGA: Polyglycolic acid
PLA: Polylactic acid
PLGA: Poly-D,L-lactic-co-glycolic acid
PLLA: Poly-L-lactid acid
PRP: Platelet rich plasma
PU: Polyuria
Scx: Scleraxis
TEC: Tissue-engineering construct
TGF-β: Transforming growth factor beta
TnC: Tenascin C
Tnmd: Tenomodulin
TSPC: Tendon stem progenitor cell
VEGF: Vascular endothelial growth factor
